# Pleiotropic actions of the male pheromone *cis*-vaccenyl acetate in *Drosophila melanogaster*

**DOI:** 10.1007/s00359-015-1020-9

**Published:** 2015-06-24

**Authors:** Aki Ejima

**Affiliations:** Department of Applied Biological Chemistry, The University of Tokyo, 1-1-1 Yayoi, Bunkyo-ku, Tokyo, 113-8657 Japan; ERATO Touhara Chemosensory Signal Project, JST, The University of Tokyo, Tokyo, 113-8657 Japan

**Keywords:** cVA, Pheromone, Courtship, Aggression, Copulation, Aggregation, Concentration, Odor context

## Abstract

The male-specific lipid, *cis-*vaccenyl acetate (cVA) has multiple functions in intra-species communication in *Drosophila melanogaster*. The presence of cVA in a male suppresses courtship motivation of other males and averts male–male courtship. Meanwhile, aggression behaviors between males are promoted by a high amount of cVA caused by increased densities of male flies. cVA also works as a modifier of courtship memory, which is suppressed courtship motivation driven by previous unsuccessful courtship experience. Conversely, cVA in the courting male stimulates female reproductive motivation and increases the probability of copulation success. It also works as an aggregation pheromone, attracting both males and females at the gathering spot. Thus, cVA is a unique example of a single molecule leading to different behaviors in response to the social context. However, despite recent advances in understanding the molecular and neural machinery for cVA sensing, it is still largely unknown how cVA triggers a specific behavior as the situation demands. In this review article, I discuss two potential machineries that might determine cVA actions for behavior selection at the sensory level.

## Introduction

Organisms are living under vast amounts of biotic and abiotic factors in habitats. Among them, they selectively receive the salient factors and adequately modulate their behaviors, leading to the gain of their survival and reproductive success. For many animals, olfaction provides critical information regarding their environmental conditions, e.g., presence of food, danger and potential mates. Message substances secreted/excreted from an individual to another conspecific one to change its behavior or physiology are called pheromones (Karlson and Luscher 1959; Shorey 1973; Wyatt 2003). In the fruit fly, *Drosophila melanogaster*, the involvement of sex pheromones for social interaction has been suggested since studies of their reproductive behaviors began (e.g., Sturtevant 1915; Jallon 1984; Ferveur 2005; Yamamoto et al. 2014). *Cis*-vaccenyl acetate (cVA) is a male-specific lipid synthesized in the ejaculatory bulb (Butterworth 1969; Brieger and Butterworth 1970; Guiraudie-Capraz et al. 2007) and has multiple functions in social behaviors including inhibition of male courtship (Jallon et al. 1981), modification of courtship memory (Ejima et al. 2007), stimulation of male–male aggression (Wang and Anderson 2010), enhancement of female copulation receptivity (Kurtovic et al. 2007), and induction of aggregation behavior (Bartelt et al. 1985). Despite recent advances in understanding the molecular and neural machinery for cVA sensing (Van der Goes van Naters 2014; Sengupta and Smith 2014; Bontonou and Wicker-Thomas 2014), how cVA, which is a single molecule, triggers different behaviors is still largely unknown.

## Multiple functions of cVA in chemical communication

### Courtship inhibition

**Fig. 1:**
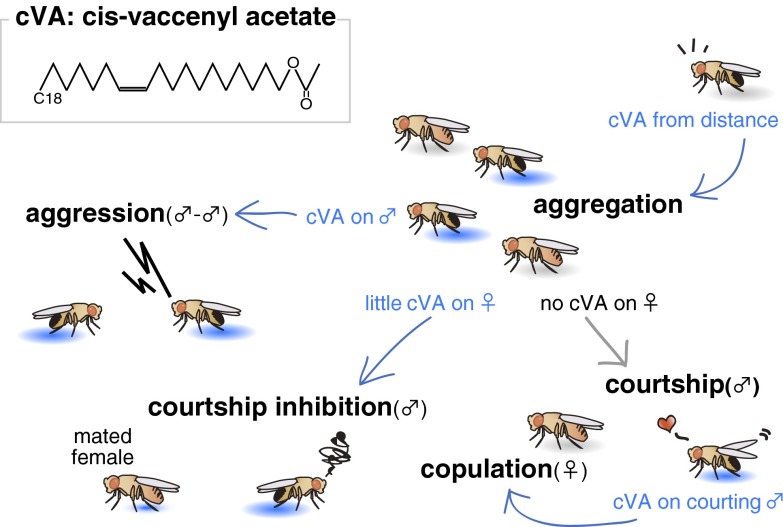
Multiple functions of *cis*-vaccenyl acetate. A male-specific lipid (Z)-11-octadecen-1-yl acetate, so-called *cis*-vaccenyl acetate (cVA) has multiple functions in social communication. From distance, cVA works as an aggregation pheromone, attracting other flies. In close proximity, for a male, presence of cVA on the other male promotes male–male aggression behavior, while absence of cVA indicates that the nearby fly is a female and stimulates courtship. Meanwhile, for a female, presence of cVA on the courting male enhances copulation receptivity. Presence of little amount of cVA on the mated female, transferred from the male during copulation, works as an anti-aphrodisiac pheromone, decreasing sexual motivation of a male

(Butterworth 1969) noticed that there is a unique lipid present in the male ejaculatory bulb and in trace amounts in the female spermathecal region after copulation. It was never detected in virgin females and he speculated that this lipid was transferred from the male to the female during copulation, and had some functions for post-mating reactions in the mated female. Soon after this male-specific lipid was identified as cVA ((*Z*)-11-octadecen-1-yl acetate (Brieger and Butterworth 1970; Fig. 1). Its biological function, however, was unclear until Jallon et al. (1981) discovered that cVA had an anti-aphrodisiac effect. They observed that either male extract or synthetic cVA inhibited male wing vibration, which is one of the typical courtship behaviors (Sturtevant 1915; Spieth 1974; Hall 1994). Application of cVA also reduced overall courtship activities (Zawistowski and Richmond 1986; Ejima et al. 2007; Kurtovic et al. 2007) and copulation success (Mane et al. 1983; Zawistowski and Richmond 1986). This anti-aphrodisiac effect of cVA is considered to contribute towards males avoiding male–male courtship.

During copulation, cVA is transferred from a male to a female (Jallon et al. 1981; Vander Meer et al. 1986; Ejima et al. 2007; Everaerts et al. 2010) and reduces the sexual attractiveness of the mated female (Tompkins and Hall 1981; Ejima et al. 2007). Because a mated female, once copulated, rarely accepts another male for about a week (Chen et al. 1988), the presence of cVA in the mated female would function as an indicator of mating status of the female and allows the male to avoid too much courtship investment on the preoccupied female. Mass spectrometry analysis of the cuticular surfaces revealed that the average amount of cVA on a single mated female 24 h after mating was 9–200 ng (Jallon et al. 1981; Vander Meer et al. 1986; Ejima et al. 2007; Billeter et al. 2009), while a single mature male possesses 200 ng to 2.9 µg cVA on its cuticular surface (Bartelt et al. 1985; Ejima et al. 2007). This means that when a male encounters another fly, he faces a challenge in distinguishing presence or absence of a tiny amount of cVA on the target fly from his self-produced cVA and also from other nearby males. It should be also noted that the amount of cVA varies over development. A newly hatched immature male contains no detectable cVA, but 4 h later, a small amount of cVA (20 ng) appears and gradually increases to reach as much as 2.9 µg at 4 weeks (Bartelt et al. 1985). Therefore, there should be some adjusting mechanism that allows the male to respond to cVA in such a variable and complex olfactory environment and leads to an appropriate behavioral decision. Further investigation into the molecular and cellular controls of the cVA responses will reveal how the male controls its olfactory sensitivity according to the context, the physiological state and/or the prior experience.

### Courtship memory

Unsuccessful courtship toward a mated female leads to gradual reduction of the male’s sexual activity (Siegel and Hall 1979). This plastic change of behavioral response is called “courtship conditioning” and has been one of the paradigms of learning and memory (Mehren et al. 2004). Considering the courtship inhibitory function of cVA per se, it was indicated that an association between the negative input of cVA and some courtship stimulatory input, e.g., female pheromone, was produced during courtship learning, and then suppressed the subsequent responses of the male. However, Ejima et al. (2005) demonstrated that interference of copulation success, not cVA, forms the courtship memory. Instead, cVA was found to be a modifier to broaden the reach of the learning effect, generalizing the saliency of the associative cue Ejima et al. (2007). Later, Keleman et al. (2012) also reported that without cVA input, the negative experience during courtship training was sufficient to manipulate subsequent male responses to the mated females.

### Aggression

When a male encounters another male, they start fighting (Chen et al. 2002; Fernández and Kravitz 2013). Wang and Anderson (2010) showed that increased cVA input through the odorant receptor OR67d is critical to promote male–male aggression at high population density (Fig. 1). Meanwhile, Liu et al. (2011) revealed that chronic exposure to cVA or group housing reduces aggression responses of the males. For this social suppression, the olfactory input through odorant receptor OR65a is critical.

Fernández et al. (2010) genetically masculinized either cuticular hydrocarbons or behaviors of the females and demonstrated that male–male aggression could occur without cVA, indicating the redundant or partial contribution of cVA to promote aggression. Agreeing with this notion, Wang et al. (2011) reported that in addition to cVA, another male factor, 7-tricosene, is necessary for a full level of aggression.

### Copulation acceptance

For a female, cVA represents maleness of the courting male and enhances her sexual motivation accordingly. As described above, the amount of cVA of a single male varies over development and, therefore, it indicates the maturation level of the male and affects female mate choice. Kurtovic et al. (2007) generated cVA-blind mutant flies and demonstrated that lack of cVA input in the females largely reduced their copulation receptivity, supporting the role of cVA input in the females.

However, the amount of cVA is not the only determinant for female mate choice. Scott et al. (2011) investigated the relationship between cVA content and the mating success of wild-caught males and found no significant correlation between the pheromonal profiles and the female’s mating preference. Male courtship song has been suggested to play an important role for a female to become receptive for copulation (Sturtevant 1915; Bennet-Clark and Ewing 1967; Hall 1994). Recently, Zhou et al. (2014) identified two clusters of the command neurons that control female receptivity and found that one of them responds to both cVA and male courtship song, suggesting a role of these neurons for information integration to promote female behavior.

### Aggregation

In addition to the roles in the reproductive behaviors described above, cVA has a non-sexual function, aggregation. Venard and Jallon (1980) demonstrated that male odor attracted both males and females in a vertical Y-maze. Using a wind-tunnel cage, Bartelt et al. (1985) showed that cVA enhanced aggregation behavior of males and females and led them to the food source. Similar results were reported by Wertheim et al. (2002) using an indoor flight cage, and Schlief and Wilson (2007) using a Y-maze. Using relatively small traps in a petri dish, Xu et al. (2005) reported cVA alone, without food, works as an aggregation pheromone in close proximity. The fact that cVA works as an enhancer of food attraction in most studies might reflect the nature of the aggregation response in the wild; the indication of the presence of conspecifics in the context of food odor might represent safety and/or quality of the food spot and accelerate food attraction. It should be also noted that males and mated females emit cVA into the environment (Bartelt et al. 1985) and this cVA deposit would attract other flies to the gathering spot even after the donor flies have left.

### Kairomone

cVA attracts other species in addition to conspecifics. Wertheim et al. (2003) discovered that a parasitoid wasp, *Leptopilina heterotoma*, which is a major predator of fly flies, is attracted by cVA and attacks fly larvae, indicating an ecological cost of cVA use. For this kairomone function, the presence of food for the flies largely enhanced the aggregation behavior of the wasps in a wind-tunnel.

## Potential underlying mechanisms for cVA pleiotropy

How is the pleiotropy of the cVA actions regulated? There are two factors to be considered, which might produce diversity of cVA function at the sensory processing level. One is input intensity, i.e., final cVA concentration when it reaches the animal’s olfactory system. The other is odor context, i.e., the olfactory background with which cVA is presented. To discuss the potential contribution of these two factors, I conducted a close inspection of the bioassay conditions of previous studies, summarized in Table 1.

**Table 1:** Experimental condition for bioassay using cVA

References	Bioassay	CVA applied	Application	Chamber size	Odor context
Jallon et al. (1981)	Courtship inhibition	220 ng			
Mane et al. (1983)	Copulation inhibition	200 ng	0.1 µl acetone	0.5 ml (0.5 cm^3^)	
Bartelt et al. (1985)	Aggregation	150 ng–150 µg		80 cm × 40 cm × 40 cm (128 × 10^3^ cm^3^)	Instant medium + yeast
Zawistowski and Richmond (1986)	Courtship inhibition	200 ng	0.1 µl acetone	0.2 cc (0.2 cm^3^)	
	copulation inhibition	100 ng	0.1 µl acetone		
Wertheim et al. (2002)	Aggregation	4.5 µg	15 µl hexane	30 cm × 40 cm × 60 cm (72 × 10^3^ cm^3^)	Mashed apple, yeast
Xu et al. (2005)	Aggregation	1 %	Unknown	10 cm × 2 cm petri dish (157 cm^3^)	
Ejima et al. (2007)	Courtship memory	0.2 ng–200 µg	Hexane	8 mm diameter × 6 mm height (0.3 cm^3^)	
	Courtship inhibition	200 µg	Hexane		
Kurtovic et al. (2007)	Courtship inhibition	10 % (≈18 ng)	0.2 µl acetone	10 mm diameter × 4 mm height (0.3 cm^3^)	
Schlief and Wilson (2007)	Aggregation	0.4 % (≈900 ng)	250 µl water	Y-maze using two 2L flasks (2 × 10^3^ cm^3^)	Propionic acid
Griffith and Ejima (2009)	Courtship inhibition	200 ng	hexane	8 mm diameter × 6 mm height (0.3 cm^3^)	Yeast paste
Wang and Anderson (2010)	Aggression	100–500 µg	Acetone	50 cm × 40 cm × 120 cm height (240 × 10^3^ cm^3^)	Apple juice + sugar
	Copulation inhibition	5 mg	Acetone		
Billeter et al. (2009)	Copulation inhibition	1 µg	Hexane	10 mm diameter × 5 mm height (0.4 cm^3^)	
Ronderos and Smith (2010)	Courtship inhibition	1 µl (≈900 ng)	(Pure)	1.5 cm diameter	
Chertemps et al. (2012)	Courtship inhibition	150 ng	Acetone	3 cm diameter × 0.5 cm height (3.5 cm^3^)	
Thistle et al. (2012)	Courtship inhibition	200 ng	Ethanol	Falcon 48-well plate (1.35 cm^3^)	Grape juice

### Concentration

Considering the nature of behavioral sequences in the wild, it would be feasible to presume that there are distinct optimum intensities/ranges of cVA input required for each behavior. For example, to attract other flies from a distance, a low amount of cVA should elicit aggregation behavior, while it should not stimulate courtship or aggression behaviors without other target flies. Wang and Anderson (2010) reported that as the number of male flies increased, the increased cVA concentration triggered the intense male–male aggression and then dispersed the group of males in the area. Therefore, a low amount of cVA indicates the presence of conspecifics and elicits approaching behavior, while, at the site where the fly encounters other individuals, a high amount of cVA indicates the sex of the target and elicits aggression behavior. Thus, the population density of the males is regulated by the push-and-pull balance between the low-cVA aggregation effect and high-cVA dispersing effect. In the case of females, a high amount of cVA represents the presence of a mature mating partner and would not have the dispersing function.

Van der Goes van Naters and Carlson (2007) reported two odorant receptors, Or65a and Or67d that respond to cVA stimulation. At the single sensillum recording, a relatively large amount of cVA was required to activate Or65a, while a small amount of cVA was enough to stimulate Or67d. It has been proposed that a distinct set of signal transduction factors, e.g., LUSH or SNMP expressed in the Or67d sensory neurons is essential for detecting the low level of cVA (Gomez-Diaz et al. 2013; Van der Goes van Naters 2014; Sengupta and Smith 2014). The distinct kinetics of the cVA responses might contribute to the concentration-dependent behavioral switch, i.e., Or67d sensory neurons, responding to low amount of cVA at a distance, lead to aggregation behavior while additional activation of Or65a neurons by high amount of cVA from a nearby fly results in aggression or courtship behavior.

Nevertheless, the sensitivity difference between Or65a and Or67d does not fully assure the behavioral switch. As described previously, interference of Or67d function alone was sufficient to suppress the acute aggression response to cVA (Wang and Anderson 2010), while the chronic suppression of Or65a neurons disturbed the social suppression of aggression (Liu et al. 2011). The temporal difference of the receptor requirement was also shown in the courtship control. Chronic suppression of Or65a neurons using tetanus toxin, but not Or67d, resulted in an impaired cVA response for courtship inhibition (Ejima et al. 2007). On the other hand, the lack of Or67d receptor was sufficient to block the acute control of courtship behavior (Kurtovic et al. 2007). Also it should be noted that the secondary olfactory neurons that receive cVA information from the Or67d neurons have sexual dimorphism in the projection pattern (Datta et al. 2008). This anatomical sexual dimorphism indicates that signaling from the Or67d neurons alone potentially convey the cues to promote the sex-specific behaviors as male–male aggression, courtship and female copulation. All together, both receptors are supposed to contribute to the behavioral responses toward a fly in close proximity. Though it should be yet determined whether the Or65 neurons are involved in the aggregation response, the underlying mechanism seems to be more complex than separated functions assigned for these two receptors.

### Odor context

As is mentioned briefly above, the power of cVA on its behavioral control differs depending on its olfactory context. For example, in addition to cVA, the presence of 7-tricosene, a cuticular component enriched in males, is essential to promote the full level of male–male aggression (Wang et al. 2011). Independently, Grillet et al. (2006) proposed the involvement of 7-tricosene in female mate choice. Therefore, it is very likely that the female copulation decision is also assured by the dual input of cVA and 7-tricosene. By extension, it is conceivable that male courtship is also controlled by the chemical context including other male or female odorants.

The long-distance aggregation function of cVA was exerted only in the presence of food odor (Table 1). The only experiment, in which cVA alone worked as an aggregation pheromone without food, was performed in a relatively small area (a 10 × 2 cm petri dish, Xu et al. (2005), compared with the wind tunnels or flight cages with air ventilation in the earlier studies (Bartelt et al. 1985; Wertheim et al. 2002). These experimental differences would explain the differences in the effective cVA concentration and the requirement of food odor as background for the behavioral output. For male–male aggression, it is known that the presence of food or female odors is required for efficient observation (Certel and Kravitz 2012). Furthermore, Griffith and Ejima (2009) demonstrated that the addition of yeast paste odor enhanced the courtship inhibitory effect of cVA, indicating that the olfactory cVA sensitivity in general is affected by the presence of food odor.

The synergistic effect of food odor has been observed also in other cVA-unrelated contexts. Grosjean et al. (2011) reported that food odorants, received by an ionotropic odorant receptor IR84a enhances male courtship. Turner and Ray (2009) found that the presence of food odor reduces CO_2_-mediated avoidance behavior, allowing the flies to stay at rotten CO_2_-emitting fruits. Altogether, a combination of multiple inputs allows an animal to fine-tune the behavioral output.
